# Single Procedure Saline Lavage for Treatment of Inspissated Bile

**DOI:** 10.1155/2020/8816599

**Published:** 2020-07-31

**Authors:** Nafisa Sideeka, Raja Shaikh, Gulraiz Chaudry

**Affiliations:** Division of Vascular and Interventional Radiology, Boston Children's Hospital and Harvard Medical School, 300 Longwood Ave, Boston, MA 02115, USA

## Abstract

Inspissated bile syndrome is a rare cause of cholestatic jaundice in infancy, occurring due to obstruction of the biliary ducts and gallbladder by biliary sludge. Traditional methods of treatment include surgical drainage or cholecystostomy drain placement. Both can be associated with complications and prolonged admission. We present 2 cases treated with a single percutaneous needle puncture of the gallbladder followed by saline lavage. Two neonates presented with cholestatic jaundice and sonographic evidence of biliary sludge and dilation of the common bile duct. Single sonographic-guided needle puncture of the gallbladder was followed by irrigation with saline. Clearing of the biliary sludge was confirmed by sonography and cholecystocholangiography. There was resolution of the cholestatic jaundice, with no complications or repeat procedures.

## 1. Introduction

Inspissated bile (IB) syndrome is a rare cause of neonatal cholestatic jaundice, with an approximate incidence of 1 in 175,000 [1]. Sonographically, this is identified as “biliary sludge” consisting of low-level motile echoes in the gallbladder and dilated biliary ducts [2]. Spontaneous resolution can occur, but if obstruction persists, open surgical drainage and irrigation or percutaneous cholecystostomy drain (PCD) placement can be required. Both can be associated with complications or requirement for prolonged drain placement [2, 3]. We present 2 cases where single percutaneous puncture of the gallbladder followed by cholecystocholangiogram and saline lavage (PCL) was sufficient to alleviate obstruction.

## 2. Case Report

At our institution, institutional review board authorization is not required for retrospective case reports.

### 2.1. Technique

The procedures were performed under general anesthesia. Both patients were receiving intravenous piperacillin-tazobactam (Zosyn), so no additional antibiotics were administered. A preprocedure sonogram was performed. Under sonographic guidance, the gallbladder was accessed percutaneously using a 21-gauge EchoTip needle (Cook Medical, Bloomington, IN). A transhepatic approach was used to minimize the risk of biliary leakage. A cholecystocholangiogram was performed, followed by 2-3 ml aliquots of normal saline flushes, with a total of 10-15 ml injected. Cholecystocholangiogram was repeated. The needle was removed, and hemostasis achieved with manual pressure. Postprocedure sonogram was performed.

**Figure 1:**
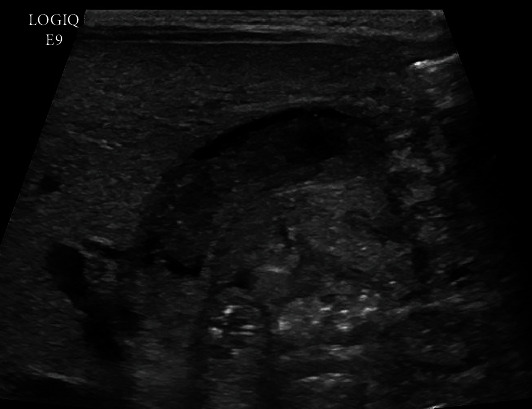
Preprocedure sonogram demonstrates echogenic sludge filling the gallbladder and CBD.

Patient 1 was a 37-week gestational age female with a birth weight of 2.7 kg with sickle cell trait and ABO incompatibility. The infant developed indirect hyperbilirubinemia requiring double volume exchange. She then developed acholic stools, with peak total and direct bilirubin of 3.6 and 2.6 mg/dl, respectively. Ultrasound demonstrated sludge in the gallbladder, intrahepatic biliary duct dilation, and dilated (5 mm) common bile duct (CBD) (Figure 1). There was no significant improvement with ursodiol.

PCL was performed at 31 days of age. Initial injection demonstrated filling defects in gallbladder and CBD. Following irrigation, spontaneous emptying of contrast was seen into the small bowel. There were no immediate complications.

Postprocedure sonogram demonstrated decompression of the biliary tract, with the CBD measuring 2 mm. The total and direct bilirubin decreased to 1.4 and 0.9 mg/dl within 1 week. Stools were noted to be pigmented. The patient was discharged 10 days postprocedure and remains asymptomatic.

Patient 2 was a 38-week gestation male with history of coarctation of aorta. At DOL 4, he underwent repair of his cardiac disease and furosemide was administered. He had progressive increase of his total and direct bilirubin, with peak of 22.3 and 4.3 mg/dl, respectively. Sonography showed sludge in the gallbladder and dilated CBD (6 mm).

**Figure 2:**
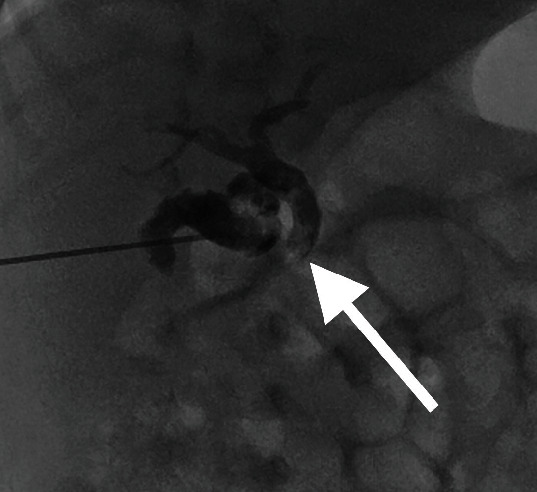
Initial cholecystocholangiogram demonstrates filling defects in the gallbladder and CBD (arrow), with evidence of obstruction and reflux into intrahepatic biliary ducts.

**Figure 3:**
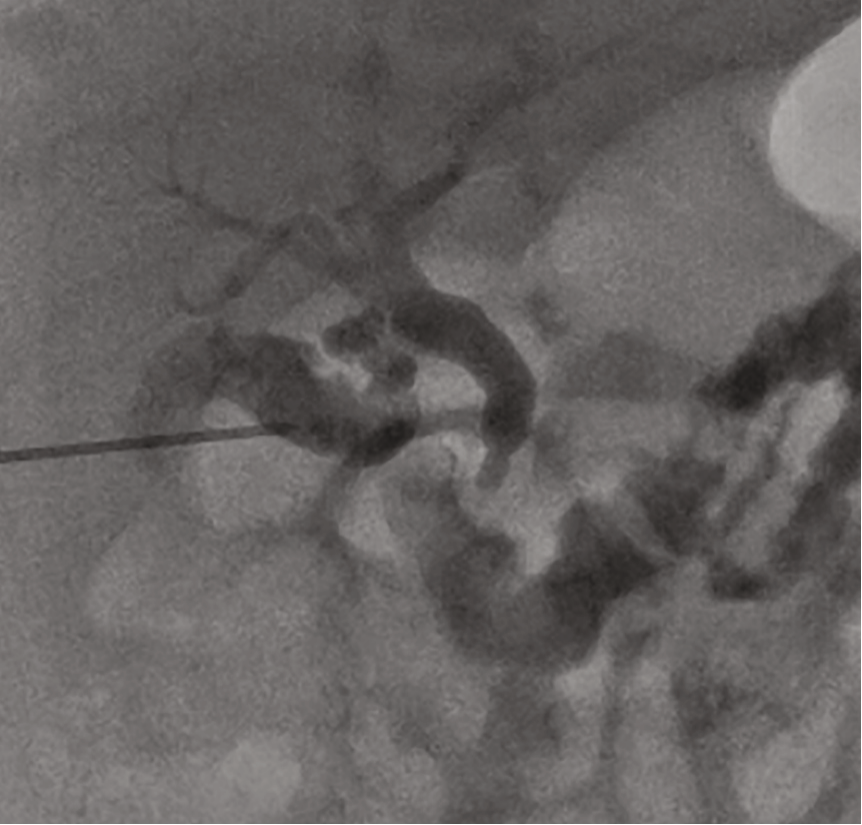
Postlavage cholecystocholangiogram shows free flow of contrast into the small bowel, with mild residual narrowing in the region of the ampulla.

Cholecystocholangiography at 17 days of age demonstrated filling defects in the gallbladder and CBD (Figure 2). Following irrigation, there was spontaneous drainage of contrast into the small bowel (Figure 3). There were no complications.

Postprocedure sonogram demonstrated near-complete resolution of the sludge and decompression of CBD (2 mm). The total and direct bilirubin decreased to 1.6 mg/dl and 1.1 mg/dl, respectively. He was discharged 9 days postprocedure with no recurrence of symptoms.

## 3. Discussion

IB as a cause of neonatal cholestatic jaundice was common in the era of Rhesus incompatibility [4]. Currently, the condition is associated with prematurity, sepsis, prolonged parenteral nutrition, and medications such as furosemide and intestinal paresis following surgery [2, 4]. Biliary sludge is composed of cholesterol monohydrate crystals and calcium bilirubinate granules suspended in strands of the gallbladder mucus [5]. Sludge formation is dependent on physical-chemical reactions of these components and associated gallbladder dysmotility [5].

Sonography demonstrates nonshadowing echogenic material in the biliary tract. The CBD is usually dilated, with variable degree of intrahepatic biliary duct dilation [3]. IB may resolve spontaneously or after the administration of ursodeoxycholic acid [6]. The degree of CBD dilation may predict the success of conservative management, with diameter greater than 4 mm likely needing intervention [7].

Historically, the intervention has been laparotomy, followed by irrigation via access into the CBD or gallbladder [8]. Davenport advocated performing percutaneous transhepatic cholangiography followed by saline flushes and proceeding to surgical intervention if that failed [4]. However, lavage of the biliary ducts alone was only successful in 8 of 14 patients with inspissated bile, with cholecystectomy or cholecystectomy required in 4 [4]. Laparoscopic cholecystostomy followed by lavage has also been described [1].

Recently, PCD has been advocated as a less invasive alternative. Pariente et al. reported placement of drains in the gallbladder and CBD for infants with cholelithiasis [9]. Helin et al. described a case of successful treatment by PCD which was then left on suction [2]. Bollu et al. demonstrated effective treatment with the use of twice daily saline flushes [3]. However, dislodgement of the catheter is a common complication of the procedure, seen in the report by Helin et al. and 3 of 7 patients by Bollu et al. [2, 3]. This sometimes necessitates a second procedure. PCD also requires prolonged admission, with drains remaining in place for a mean of 26 days in the study by Bollu et al. [3].

Sonographic-guided percutaneous cholecystocholangiogram is a simple technique with very low complication rate and is sometimes used to diagnose biliary atresia in infants [10]. Contrast is injected through a small caliber needle to outline the gallbladder and biliary tract. As seen in our patients, irrigation with saline through this small caliber access may be enough to resolve the obstruction. If there is evidence of residual obstruction, the same access can be used to place a pigtail drain. Access of the gallbladder, rather than the biliary ducts, allows clearance of sludge from the gallbladder and cystic duct. Potential complications include bleeding, biliary peritonitis, and infection but were not seen in our patients.

It is difficult to determine the efficacy of the procedure based on 2 cases. In addition, it can only be performed in patients without an underlying anatomic abnormality of the biliary system. It is possible that if the underlying process is not resolved, there may be recurrence of obstruction requiring a repeat procedure.

In conclusion, in neonates with inspissated bile, percutaneous cholecystocholangiography followed by saline lavage may be sufficient to resolve obstruction, potentially obviating the need for cholecystostomy drain placement or surgical intervention.
